# Effect of Freezing on Photosystem II and Assessment of Freezing Tolerance of Tea Cultivar

**DOI:** 10.3390/plants8100434

**Published:** 2019-10-22

**Authors:** Yun-Long Shi, Zhuo-Yu Cai, Da Li, Jian-Liang Lu, Jian-Hui Ye, Yue-Rong Liang, Xin-Qiang Zheng

**Affiliations:** Tea Research Institute, Zhejiang University, Hangzhou 310058, China; lonfucius@yahoo.com (Y.-L.S.); 21716160@zju.edu.cn (Z.-Y.C.); 21616098@zju.edu.cn (D.L.); jllu@zju.edu.cn (J.-L.L.); jx0515@163.com (J.-H.Y.)

**Keywords:** *Camellia sinensis*, freezing tolerance, freezing sensitivity, overwintering, photosynthesis system, tea breeding

## Abstract

Freezing tolerant tea cultivars are urgently needed. The tea cultivars with highly freezing tolerance showed resistance to freezing stress induced photoinhibition. Freezing sensitivity index (H) of 47 tea clonal cultivars was investigated after severe freezing winter in 2016. To develop instrumental methods for freezing tolerance selection, the maximum photochemical efficiency of photosystem II (PSII) (Fv/Fm) and leaf color indicator *a* on the Hunter color scale were determined on control group (non-frozen) and frozen group (being frozen at −15 °C for 2 h and then stood at 20 °C for 5 h) of the cultivars. When the two indicators were expressed as the ratios (R_Fv/Fm_ and R*_a_*) of frozen group to control group, linear regression of the freezing sensitivity index (H) upon the R_Fv/Fm_ and R*_a_* produced significant relationship respectively, i.e., H = 60.31 − 50.09 R_Fv/Fm_ (*p* < 0.01) and H = 30.03 − 10.82 R*_a_* (*p* < 0.01). Expression of gene *psbA* encoding D1 protein and gene *psbD* encoding D2 protein in PSII showed that the frezzing tolerant tea cultivars maintained a high expression level of *psbA* after freezing stress, which is considered to be beneficial to *de novo* synthesis of D1 protein and sustaining PSII activity. These findings can provide instrumental tools for assessing freezing tolerance of tea cultivars in tea breeding program.

## 1. Introduction

Low temperature affects the growth, development, and productivity of tea plants, and severe freezing winter conditions at high latitudes limit the distribution of tea plants and the expansion of tea production areas. However, there are obvious differences in freezing tolerance between tea cultivars [1]. For example, tea cultivar ‘Yinghong 1’, bred from descendants of *Camellia assamica* could not survive in the lower reaches of Yangtze River around 30° N latitude where the lowest winter temperature is about −6 °C, whereas cultivar ‘Lucha 1’ bred from descendants of *Camellia sinensis* grows well in Qingdao City, China where the latitude is 36° N and the lowest winter temperature is below −10 °C. Breeding and cultivating freezing tolerant tea cultivars is an effective way to conquer freezing stress in winter at high latitude tea areas.

Assessing the freezing tolerance of tea cultivar or tea line from the tested hybrid population or tea lines is a key technique for breeding freezing tolerance tea cultivar. To accelerate the freezing tolerance breeding, various indicators were introduced to evaluate the freezing tolerance potential of the tested tea cultivar. These indicators included the layers of palisade cell and the ratio of the palisade to spongy parenchyma in leaf transverse section [2], leaf electrolyte leakage after freezing treatment [3]. Transcriptome sequencing was also used to explore genes or DNA markers linked to cold tolerance of tea cultivar [4,5,6,7,8]. However, there is a long way to go for applying these indicators in tea breeding practices. Actually, field survey is still an indispensable method to evaluate the freezing tolerance of the tested tea lines, in which freezing-induced injuries of various tea accessions grown in the same field were assessed after severe freezing weather in winter. However, this operation is highly weather dependent and time consuming. The freezing tolerance assessment could not be carried if the winter was warm. It is necessary to develop feasible instrumental methods for assessing the freezing tolerance of tested tea cultivars to guide freezing resistance tea breeding.

Various ecotypes of crops showed distinct differences in their potential to acclimate to cold and hot stress [9]. Chloroplast, a photosynthesis site in a plant, is one of the first organelles affected by cold stress and it extremely sensitive to cold stress [10,11]. Chloroplast proteolytic machinery is involved in repairing damage incurred to the photosynthetic machinery upon exposure to cold stress [12]. The cold-tolerant ecotype of *Valonia utricularis* is able to cope with the applied low temperature stress by down-regulating the PSII RCs, whereas the cold-sensitive ecotype has a generally lower capacity for dynamic photoinhibition and sustains photodamage [13]. In rice, cold tolerance is connected to photosynthesis regulation [14]. The chilling-induced differences in chloroplast structure between cold sensitive and cold tolerant plants are the consequence of different thylakoid supercomplex rearrangements [15]. The PSII protein complex contains more than 20 subunits and includes both extrinsic and intrinsic membrane proteins [16]. Some intrinsic proteins including D1 protein encoded by gene *psbA* and D2 protein encoded by gene *psbD* are required for phototroph growth and oxygen evolution. The deletion of these proteins causes disruption of the PSII assembly and function. The subunit structure of the intrinsic proteins has been highly conserved in higher plants and is necessary to allow oxygen evolution, while the extrinsic proteins have undergone a large evolutionary change [17]. PSII proteins can undergo posttranslational modifications associated with stress protection and repair and the PSII intrinsic proteins in cold tolerant plants have strong repair ability to prevent the photosynthetic system from the chilling-enhanced photooxidation and maintain a high photosynthetic capacity [16,18]. There is connection between cold tolerance and photosynthesis regulation in plant.

The freezing tolerance of crops was indirectly assessed by detecting their photosynthetic activity under freezing stress. Chlorophyll fluorescence (ChlF) and potential quantum yield of PSII (Fv/Fm), which characterize the activity of PSII RCs, were used as selection tools for improving the cold tolerance of crops such as maize, tomato and rice, which was largely due to the ability of the tolerant plants to keep higher efficiency of excitation energy capture by the PSII RCs [19,20,21,22,23]. The information on the relationship of photosynthetic regulation to freezing tolerance of tea cultivars has not been available.

Tea leaf is rich in tea polyphenols (TP) which are suspended in the leaf cell vacuoles. When the leaves are injured by mechanical action or environmental stress such as freezing or chloroform vapor, the TP in the leaves will be oxidized, resulting in browning of the leaves [24]. It is hypothesized that the changes of the leaf color depend on the severity of leaf injury and those from freezing sensitive cultivars will become more red brown owing to their serious damage induced by the freezing. Therefore, the present study is aimed to investigate the effects of freezing on the PSII activity and leaf color of various tea cultivars, so as to search a tool for screening freezing tolerant tea lines in tea breeding program.

## 2. Results

### 2.1. Freezing Tolerance of Various Tea Cultivars

The field survey results showed that among the forty seven tested cultivars, four was rated as freezing sensitive, with an H index ≥ 50.00. Thirty-five was rated as moderate freezing sensitive, with H indexes ranging from 20.00 to <50.00. One was rated as freezing tolerant, with an H index 18.33, and seven was rated as highly freezing-tolerant, with H indexes < 10.0 (Table 1). These suggest that there were obvious differences in freezing tolerance between various tea cultivars and cultivating tea cultivars with freezing tolerance will be an affective measure to conquer freezing winter weather in high latitude tea areas.

### 2.2. Effects of Freezing on Fv/Fm of Leaves of Various Tea Cultivars

Fv/Fm is an indicator of the maximum photochemical efficiency of PSII and it can be used to assess the PSII activity [25]. The Fv/Fm was determined from the ratio of variable (Fv) to maximum (Fm) fluorescence (Fv/Fm = (Fm − F_0_)/Fm) [26]. Table 2 shows that the mean value of Fv/Fm was significantly decreased from 0.745 to 0.526 (*p* < 0.001). The mean value of Fv/Fm ratio of frozen group to control group was 0.706, suggesting that 29.4% of the maximum photochemical efficiency of PSII was lost after freezing treatment, in which there were obvious differences in the freezing-induced decrease in Fv/Fm value between tea cultivars, ranging from a decrease by 66.3% (freezing sensitive cultivar ‘5-62’) to a decrease by 4.2% (highly freezing tolerant cultivar ‘4-52’) (Table 1 and Table 2). These suggest that the responses of various tea cultivars to the freezing-induced decrease in Fv/Fm were differentiated, in which it decreased more in freezing sensitive cultivars than highly freezing-tolerant cultivars.

Statistics showed that the Pearson’s linear correlation coefficient (r) between freezing sensitivity index (H) and Fv/Fm ratio of frozen group to control group (R_Fv/Fm_) was −0.610 (*p* < 0.01), with a linear regressive relationship as H = 60.31 − 50.09 R_Fv/Fm_ (*p* < 0.01, n = 47) (Table S1), suggesting that the freezing tolerance of a tea cultivar can be predicted by its R_Fv/Fm_ value.

### 2.3. Effects of Freezing on Expression of Genes Involved in PSII Proteins D1 and D2

**Figure 1:**
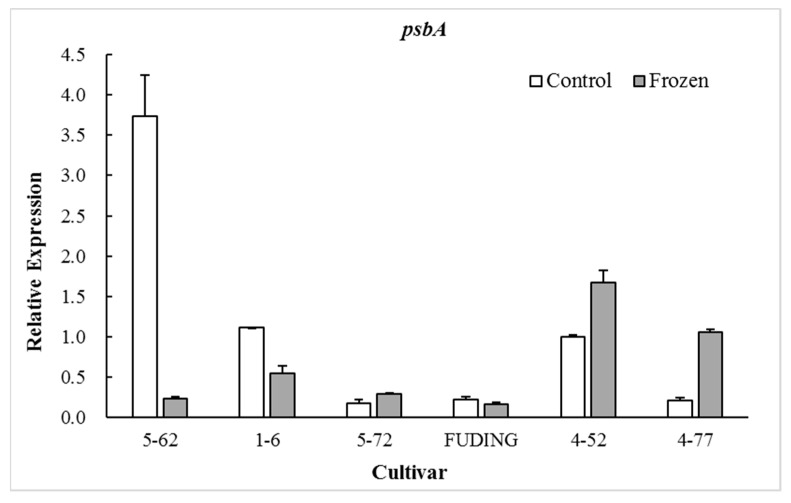
Effect of freezing on the expression of gene *psbA*.

**Figure 2:**
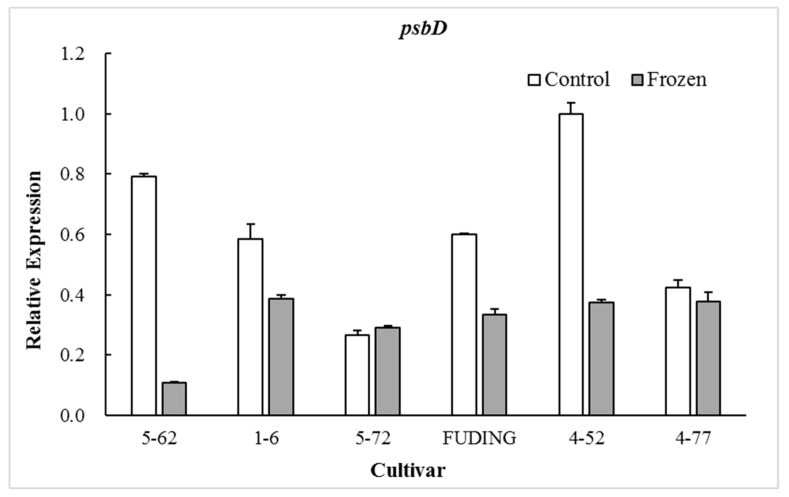
Effect of freezing on the expression of gene *psbD*.

The photosynthetic proteins play an important role in preventing PSII from photodamage under environmental stress conditions [27,28]. Among the various subunits in the PSII reaction center (RC), D1 protein encoded by chloroplast *psbA* is a major target that is easily injured by environmental stress [29]. The timely synthesis of new D1 protein is key to PSII functional recovery [30] and D2 protein encoded by *psbD* gene is closely related to the repair of D1 protein [31]. Therefore, the transcription of the *psbA* and *psbD* genes plays an extremely important role in the process of D1 protein turnover and PSII function during environmental stress conditions [32]. The present study showed that the expression of *psbA* and *psbD* in the freezing sensitive cultivar ‘5-62’ was significantly decreased by freezing treatment (*p* < 0.05). However, the expression patterns of these two genes in highly freezing-tolerant cultivars such as ‘4-52’ and ‘4-77’ were differentiated by freezing treatment, in which *psbA* was significantly upregulated (*p* < 0.05) while *psbD* was downregulated. There were no significant differences in expression of *psbA* in moderate freezing-sensitive cultivars ‘5-72’ and ‘Fuding’ after freezing treatment (Figure 1). The freezing-induced upregulation of *psbA* gene in the highly freezing-tolerant tea cultivars might be beneficial to the balance of D1 protein and the PSII functional recovery during freezing stress. No obvious relationship was observed between the cold sensitivity index H and the expression level of *psbD* (Figure 2).

When calculating the relative expression values, the tested data were firstly normalized using *β-actin* as an internal reference, and then made relative to the control of ‘4-52’ in which the expression value of ‘4-52’ control was fixed as 1.0.

When calculating the relative expression values, the tested data were firstly normalized using *β-actin* as an internal reference, and then made relative to the control of ‘4-52’ in which the expression value of ‘4-52’ control was fixed as 1.0. 

### 2.4. Effects of Freezing on Leaf Color of Various Tea Cultivars

When values on Hunter color scales were used as color indicators, it was showed that the light-dark scale *L* value and green-red scale *a* value were significantly increased, whereas the blue-yellow scale *b* value significantly decreased after freezing treatment (Table 3). The *L* is a white-black indicator, in which the sample would be a perfect reflecting diffuser when the *L* is at maximum value 100, and it would be black when the *L* is at minimum value 0. The *a* value is a green-red indicator, in which a positive *a* represents red color and a negative *a* represents green color. The *b* value is a blue-yellow indicator, in which a positive *b* suggests the sample is in yellow color and a negative *b* suggests the sample is in blue color [33]. The increase in the L value of the frozen group suggests that the color of the frozen leaves became lighter than the leaf color of control group after freezing treatment. The increase of value *a* from −11.66 to −6.27 in the frozen group suggests that the frozen leaves were less green in color than the control leaves. These might partially be due to the freezing-induced degradation of leaf photosynthetic pigments such as chlorophylls, and partially be due to the oxidation of foliar TP. The decrease in the *b* in the frozen leaves implies the frozen leaves became less yellow in color, which might be due to the loss of yellow pigments such as carotenoids.

Analysis of the Pearson’s linear correlation coefficient (r) showed that there was a significantly negative correlation between the freezing sensitivity index (H) and the ratio of frozen group *a* to control group *a* (R*_a_*) (r = −0.480, *p* < 0.01, n = 47), with a linear regressive relationship as H = 30.03 − 10.82 R*_a_* (*p* < 0.01) (Table S1), suggesting that the freezing tolerance of a tea cultivar can be predicted by its R*_a_* value. The freezing sensitivity index H was not significantly correlated to the indicators R*_L_* and R*_b_* (Table S1).

## 3. Discussion

Distribution of tea cultivars depends on their freezing tolerance. Cultivating tea cultivars with high freezing-tolerance is very important for the high latitude tea areas where the winter is severely cold. Tea plants were originated in tropical mountainous areas and they were usually sensitive to freezing environments, especially in the areas with severe winters. With the continuous expansion of tea planting areas to the high latitude areas and the artificial selection of cultivars, the freezing tolerance of tea plants or cultivars have been differentiated genetically [7]. Table 1 reveals that the tea cultivars with various freezing tolerances showed normal distribution rules, i.e., most of the cultivars showed moderate freezing-tolerant, while a few showed highly freezing sensitive or freezing-tolerant, being consistent with the previous reports [34,35]. It is feasible to screen some tea plant individuals or lines from the existing tea germplasm resources or hybrids. However, the manual selection based on field surveys depends on weather conditions and it will take several years. It is necessary to develop objective instrumental methods for screening lines or plant individuals with high freezing-tolerance from the existing breeding materials (hybrid descendants, radiation and chemical induced mutants and natural mutants).

Freezing stress severely affects the growth and development of plants. The present study showed that there were obvious differences in freezing tolerance between tea cultivars (Table 1), and these differences could be identified according to the freezing-induced changes in Fv/Fm of the tea leaves (R_Fv/Fm_) (Table 1 and Table 2). Based on these findings, the present paper reveals a linear regressive relationship between freezing sensitivity index (H) and Fv/Fm ratio of frozen group to control group (H_Fv/Fm_): H = 60.31 − 50.09 R_Fv/Fm_ (*p* < 0.01) (Table S1). Chloroplast is extremely sensitive to low temperature stress and also one of the first organelles affected by suboptimal growing temperature [11]. Low temperature stress induced thylakoid super-complex rearrangements, resulting in differentiation of chloroplast structure between cold sensitive and cold tolerant plants [15]. Chilling induced changes in the grana thylakoid fluidity could distort the balance of photosystem rearrangements, resulting in sensitivity of cucumber to chilling [10]. Various ecotypes (*Valonia utricularis*) were differentiated in their potential to acclimate to suboptimal growth temperatures [9]. Cold stress led to reductions in photosynthetic rate in *Elymus* [36]. The primary freezing-triggered damage could be partially repaired, but further freezing-triggered dysfunction of the electron transfer between the PSII RCs and the primary quinone electron acceptor of PSII (QA) was connected with secondary damage leading to PSII deactivation. The Fv/Fm, an indicator that characterizes the efficiency of energy trapping in the PSII RCs, was different between the cultivars which had various freezing-tolerance capacities during the recovery stage [21]. The cold-tolerant ecotypes (*Miscanthus* and *Saccharum*) were able to cope with applied low temperature stress by down-regulating PSII RCs, whereas the cold-sensitive ecotypes had a generally lower capacity for dynamic photoinhibition and sustain photodamage [37]. The strong cold-tolerance of sorghum PSII to photo-inactivation was because of its more effective dissipation capacity of the excess of energy and to a more balanced diversion of the absorbed energy into alternative energy sinks [38]. In winter, the cold resistant tea cultivar always contains higher level of soluble carbohydrates than the cold sensitive cultivar, resulting in lower freezing point temperature in the former than that in the later [35]. During cold stress, cold-tolerant accessions of *Arabidopsis thaliana* maintain higher PSII activity and higher capacity for sucrose synthesis than the cold sensitive accessions [39]. That is why cold resistant inbred lines of crops could be screened by analyzing the ChlF under cold stress [19,20,22].

The proteins D1 and D2 in chloroplast are two key subunits in the PSII RCs and they are present in association with chlorophyll and pheophytin [40]. The D1/D2 heterodimer may serve a similar function in PSII in a plant to that of the L/M pair in the bacterial reaction center [41]. The D1 protein is a key target of environmental stress [29]. The present study shows that the expression of gene *psbA* encoding D1 protein in freezing sensitive tea cultivars ‘5-62’ was greatly downregulated whereas that in highly freezing-tolerant tea cultivars ‘4-52’ and ‘4-77’ was significantly upregulated after freezing treatment, with moderate freezing-sensitive cultivars ‘5-7’ and ‘Fuding’ in between showing less change (Figure 1). This is consistent with the findings in *Zea mays* and *Colobanthus quitensis* that the cold tolerant genotypes were able to maintain D1 protein level after photoinhibitory conditions by upregulating *psbA* mRNA levels for the D1 biosynthesis [42,43]. The involvement of D2 subunit in the responses to low temperatures may vary with plant species. A study on lotus species showed that the D2 subunit was more severely affected by low temperature exposure than D1 [44]. However, this study showed no obvious relationship between the *psbD* expression and freezing tolerance of tea cultivars (Figure 2). These results suggest that the freezing-induced decrease in Fv/Fm of the freezing sensitive tea cultivar might be a consequence of its weak dynamic capacity to balance the synthesis and loss of D1 protein. Analysis of the expression of gene *psbA* encoding the D1 is considered to be used as a selection tool for freezing tolerance of photosynthesis in tea breeding. 

Furthermore, the agronomic measures that could promote the expression of *psbA* will be beneficial to enhance the freezing tolerance of crops. There was a study showing that the gene *WHY1* binding to the upstream region of *psbA* enhanced the cold tolerance of tomato by increasing the de novo synthesis of D1 protein and maintaining the function of PSII in chloroplasts [45]. The application of chitooligosaccharides could enhance cold tolerance of rice by repairing the photodamaged PSII through upregulating *psbA* [46]. This technique is worth trying in the high latitude tea growing areas with severely freezing winter.

Tea leaf contains both TP and polyphenol oxidase (PPO). In the intact leaf, the TP which is suspended in the vacuoles is separated from the PPO that is bound to the thylakoid membrane. However, they will contact each other when the protoplasmic membrane systems were damaged by environmental stress, in which the PPO will catalyze the TP oxidation, resulting in the changes in leaf color from green to red brown. In selection of black tea cultivars, fresh tea leaves of various tested hybrid plants or clonal cultivars were placed in a sealed container with chloroform for e few hours, during which the protoplasmic membrane system of the leaves was injured by the chloroform vapour and the TP was oxidized, and then the fermenting abilities of the tested tea cultivars were assessed according to the redness of the leaves [24]. In the present study, the leaves on tea shoots were frozen at −15 °C for 2 h and then stood at room temperature for 5 h, during which the protoplasmic membrane systems in leaves on the freezing sensitive cultivars with high freezing point temperature were injured more seriously than the freezing tolerant cultivars, resulting in differentiation in TP oxidation and changes in the “green-red” indicator *a* on the Hunter color scale. That is why the “*a*” ratio of the frozen group to the control group (R*_a_*) showed significantly regressive relationship to the freezing sensitivity index H (H = 30.03 − 10.82 R*_a_*, *p* < 0.01). This will provide a tool for instrumental screening freezing tolerant tea plants from a population of hybrid plant individuals.

## 4. Materials and Methods

### 4.1. Plant Materials

Plant materials used in the present paper were forty seven tea clonal cultivars with 8-year-old, grown in Tea Farm of Zhejiang University, Hangzhou, China (120.30° E, 30.42° N). The tea plants were planted in random block design, in which 15 plants in each block, with three replicates. On 24 January 2016, the lowest temperature was −10 °C (Figure S1), which was the lowest temperature since 2011. The plants of various tea cultivars showed obvious differences in the freezing-induced injuries with the temperature increase. The freezing-induced injuries of these cultivars were differentiated in the middle of February 2016 when the temperature increased to 20 °C or above (Figure S2). The field survey was carried out on 15 February 2016.

### 4.2. Investigation of Freezing-Induced Injuries

The plants (5 plants each block) for freezing-induced injuries investigation were randomly chosen, and then the healthy leaves and the freezing-induced injured leaves on the plants were counted separately. The freezing sensitivity index (H) was calculated by the following equation [47]: H (%) = [Σ(n*_i_* × x*_i_*)/(N × 4)] × 100.

Where the H is freezing sensitivity index, n*_i_* is the number plant with a freezing-sensitive score *i* in the five grading system (Table S2), x*_i_* is the freezing-sensitive score *i* in the five grading systems, N is the total plant number of the investigated cultivar, 4 is the highest score of *i* in the five grading system (Table S2). The freezing tolerance of a cultivar was evaluated based on the H value (Table S3): highly freezing-tolerant (H < 10.0), freezing-tolerant (10.0 ≤ H < 20.0), moderate freezing-sensitive (20.0 ≤ H < 50.0) and freezing-sensitive (H ≥ 50.0) [47].

### 4.3. Freezing Treatment

#### 4.3.1. Effect of Freezing Temperature on Changes in Leaf Color

The green color of fresh tea leaves will change into brown color when the protoplasmic membrane system in the leaf cells is damaged by stress conditions such as chloroform vapor, heating and freezing due to the oxidation of foliar TP [24]. To find an optimum temperature to treat the tea shoots for assessing the freezing tolerance of various tea cultivars, 25 tea shoots with an apex bud and six leaves were cut from each tea cultivar (cultivars ‘FZ-0’, ‘1-17’, ‘4-63’, and ‘1-10’) and then randomly divided into five groups (5 shoots each). One group (5 shoots) was placed in a 500-mL beaker with 200 mL purified water at 20 °C (control group). The other four groups (five shoots each) were placed in refrigerators setting at −5, −10, −15, and −20 °C for 2 h, respectively, and then were took out and placed in 500-mL beakers containing 200 mL purified water at 20 °C as the control group, during which the changes in leaf color were observed every hour. The observation showed that little change in leaf color for treatments at −5 and −10 °C. The color of the frozen leaves treated at −15 and −20 °C changed obviously from second hour after placing in the 500-mL beaker containing water and the colors were stabilized after 5 h (Figure S3). Therefore, in the subsequent freezing treatment, the tea shoots were frozen at −15 °C for 2 h and then stood in 500-mL beaker containing 200 mL purified water for 5 h before Fv/Fm testing and colorimetric testing. 

#### 4.3.2. Freezing Treatment of Various Tea Cultivars

Based on the above results, the control group (5 shoots each cultivar) was treated as before, and the tested group (5 shoots each cultivar) of shoots with an apex bud and six leaves were frozen at −15 °C for 2 h and then stood in 500-mL beaker containing 200 mL purified water for 5 h at room temperature before Fv/Fm testing and colorimetric testing, in which the fourth leaves beneath apex bud of the frozen and control groups were picked for testing.

### 4.4. Fv/Fm Testing

The maximum photochemical efficiency of PSII (Fv/Fm, the ratio of variable (Fv) to maximum (Fm) fluorescence (Fv/Fm = (Fm − F_0_)/Fm) in the frozen and control groups of each cultivar was determined using a Handy PEA fluorimeter (Plant Efficient Analyzer, Hansatech Instruments Ltd., King’s Lynn, Norfolk, UK). The leaves were dark-adapted for 20 min before measurement. The values of Fv⁄Fm were read on the Handy PEA fluorimeter [25], which were used to assess the PSII activity. Five leaves were tested for each treatment.

### 4.5. Test of Expression of Genes psbA and psbD

The expression of gene *psbA* encoding D1 protein and gene *psbD* encoding D2 protein was tested by real-time quantitative PCR (rt-qPCR). The extraction of total RNA and synthesis of cDNA were carried out by a previous method [48]. The rt-qPCR primers of *psbA* (genome ID: TEA001596.1) and *psbD* (genome ID: TEA011225.1) (Table S4) were designed according to the published reference genome [49]. The rt-qPCRs were conducted on a StepOne Plus Real-Time PCR system (Applied Biosystems, Foster City, CA, USA) using PowerUp^TM^ SYBR^TM^ Green Master Mix (Applied Biosystems, Foster City, CA, USA) for the detection of PCR products. Each reaction was performed in a final volume of 15 μL, including 5.4 μL sterile deionized water, 7.5 μL SYBR Mix, 0.45 μL primer (600 nM ) and 1.2 μL cDNA template (60 ng). The thermal cycling conditions were 95 °C for 2 min, followed by 40 cycles of 95 °C for 3 s, 60 °C for 30 s with fluorescence detection at the end of each cycle and confirmation by melting curve analysis. The rt-qPCR test was generated using triplicate leaf samples and each sample was tested in technical triplicates. Each relative expression level was calculated using the 2^−ΔΔCT^ method with *β-actin* gene as internal control [50]. The relative expression values were firstly normalized using *β-actin* gene as an internal reference. For plant materials for rt-qPCR, totally six clonal tea cultivars were chosen from the groups of highly freezing-tolerant, moderate freezing-sensitive and freezing-sensitive cultivars, respectively.

### 4.6. Colorimetric Testing

Indicators of *L*, *a* and *b* on the Hunter color scales for the leaves of the frozen group and control group were determined using an UltraScan VIS1195 HunterLab colorimeter (Hunter Associates Laboratory, Inc., Reston, VA, USA) according to the guidelines of the instrument.

### 4.7. Statistic Analysis

The biological repeat was 3 (blocks) and technical repeat was 5 (5 plants each block) in this study. Analysis of weighted means, standard deviation, Pearson’s linear correlation coefficients, and linear regression was carried out on software IBM SPSS Statistics V20.

## 5. Conclusions

The freezing tolerances of tea cultivars are differentiated as normal rule distribution, in which a few are highly freezing-tolerant and freezing sensitive respectively, with most of them being moderate freezing-sensitive. Traditionally, freezing-tolerant tea plants were screened from a big population of hybrid tea plants after severely freezing weather, and this operation took a few years but the selection effectiveness was dependent on the weather conditions. Developing a feasible instrumental method for screening freezing-tolerant plant individuals from the tested breeding population will be significant for breeding highly freezing-tolerant tea cultivars. When tea shoots with six leaves and a bud were frozen at −15 °C for 2 h and then stood at room temperature (20 °C) for 5 h, the maximum photochemical efficiency of PSII (Fv/Fm), the expression of gene *psbA* encoding D1 protein in PSII CRs and leaf color indicator *a* on the Hunter color scale were obviously differentiated between tea cultivars with various freezing tolerances, among which the Fv/Fm ratio of the frozen group to control group (R_Fv/Fm_) and the *a* ratio of frozen group to control group (R*_a_*) showed significantly linear regressive relationship to the freezing sensitivity index (H) obtained by field surveys after severe freezing weather, with H = 60.31 − 50.09 R_Fv/Fm_ (*p* < 0.01) and H = 30.03 − 10.82 R*_a_* (*p* < 0.01). These will provide instrumental tools for the assistant selection of freezing tolerant cultivars in the tea breeding program.

## Figures and Tables

**Table 1:** Percentage of freezing-induced injured leaves of various cultivars ^1^.

Cultivars	Block 1 (%)	Block 2 (%)	Block 3 (%)	Mean ± SD (%)	H	Freezing Tolerance
4-56	16.06	18.32	17.40	17.263 ± 1.138	50.00	Sensitive
5-62	21.83	22.81	23.46	22.701 ± 0.817	50.00	Sensitive
FZ-0	16.08	16.75	17.40	16.741 ± 0.660	50.00	Sensitive
HJY	20.51	23.08	23.06	22.217 ± 1.478	50.00	Sensitive
1-17	15.89	16.23	16.50	16.204 ± 0.304	48.33	Moderate sensitive
4-57	14.91	16.95	16.97	16.278 ± 1.184	48.33	Moderate sensitive
Zijuan	15.41	13.79	16.71	15.303 ± 1.465	43.33	Moderate sensitive
4-63	14.23	14.08	14.22	14.178 ± 0.083	33.33	Moderate sensitive
1-16	13.78	13.36	12.95	13.364 ± 0.417	28.33	Moderate sensitive
1-6	14.55	13.17	12.11	13.279 ± 1.226	28.33	Moderate sensitive
JK-2	13.93	13.66	13.95	13.847 ± 0.164	28.33	Moderate sensitive
4-14	13.29	14.74	13.37	13.801 ± 0.815	26.67	Moderate sensitive
2-49	8.81	9.46	9.14	9.139 ± 0.326	25.00	Moderate sensitive
2-7	6.99	6.74	6.72	6.815 ± 0.150	25.00	Moderate sensitive
3-1	13.25	13.58	13.00	13.273 ± 0.291	25.00	Moderate sensitive
3-10	11.86	12.49	13.01	12.456 ± 0.576	25.00	Moderate sensitive
4-152	8.75	8.83	8.85	8.814 ± 0.054	25.00	Moderate sensitive
4-154	9.49	8.72	8.41	8.876 ± 0.557	25.00	Moderate sensitive
4-17	8.12	7.73	7.36	7.738 ± 0.381	25.00	Moderate sensitive
4-38	9.23	8.31	8.32	8.620 ± 0.532	25.00	Moderate sensitive
4-44-4	12.4	12.03	12.16	12.195 ± 0.187	25.00	Moderate sensitive
4-45	9.58	9.47	8.73	9.257 ± 0.462	25.00	Moderate sensitive
4-6	7.55	8.51	8.35	8.139 ± 0.513	25.00	Moderate sensitive
4-76	12.89	12.43	13.06	12.790 ± 0.325	25.00	Moderate sensitive
5-47	9.89	9.41	9.06	9.454 ± 0.419	25.00	Moderate sensitive
5-72	10.82	10.40	10.37	10.532 ± 0.252	25.00	Moderate sensitive
5-81	10.77	10.40	10.30	10.489 ± 0.245	25.00	Moderate sensitive
FZ-1	11.59	13.10	13.02	12.568 ± 0.846	25.00	Moderate sensitive
FZ-2	8.78	9.32	9.13	9.076 ± 0.272	25.00	Moderate sensitive
PBZY	10.61	10.51	10.03	10.384 ± 0.309	25.00	Moderate sensitive
ZNB	10.20	10.72	10.24	10.385 ± 0.288	25.00	Moderate sensitive
Fuding	11.93	11.94	11.75	11.875 ± 0.106	25.00	Moderate sensitive
Fuwuming	7.93	8.54	8.62	8.367 ± 0.377	25.00	Moderate sensitive
Jinxuan	10.35	10.45	10.07	10.29 ± 0.195	25.00	Moderate sensitive
Xiangshan 3	7.80	8.05	7.91	7.921 ± 0.126	25.00	Moderate sensitive
Xiangshan 5	9.59	10.35	10.44	10.127 ± 0.463	25.00	Moderate sensitive
Zhenghe	7.48	8.37	7.66	7.838 ± 0.467	25.00	Moderate sensitive
1-18	7.96	7.51	6.30	7.255 ± 0.859	23.33	Moderate sensitive
Z-7	4.90	5.97	5.48	5.450 ± 0.532	20.00	Moderate sensitive
5-28	5.19	5.42	5.09	5.229 ± 0.169	18.33	Tolerant
1-35	4.53	4.00	2.97	3.832 ± 0.792	1.67	Highly tolerant
1-10	0.22	0.21	0.20	0.209 ± 0.006	0	Highly tolerant
4-52	0.24	0.59	0.58	0.469 ± 0.198	0	Highly tolerant
4-52-2	0.45	0.48	0.44	0.457 ± 0.022	0	Highly tolerant
4-77	0.67	0.83	0.56	0.687 ± 0.136	0	Highly tolerant
FYWM-3	1.77	2.16	1.37	1.768 ± 0.395	0	Highly tolerant
FYWM-7	2.42	2.66	2.48	2.519 ± 0.126	0	Highly tolerant

^1^ The freezing-induced injured leaves and total leaves of 5 plants each block was counted and the percentage of the freezing-induced injured leaves was expressed as (total injured leaves/total leaves) × 100%. The H index was calculated by equation (H (%) = [Σ (n*_i_* × x*_i_*)/(N × 4)] × 100) described in Section 4.2.

**Table 2:** Effects of freezing on Fv/Fm in leaves of various tea cultivars.

Cultivars	Frozen Group	Control Group	R_Fv/Fm_^1^
1-10	0.642 ± 0.055	0.732 ± 0.023	0.877
1-16	0.400 ± 0.055	0.749 ± 0.025	0.534
1-17	0.493 ± 0.126	0.709 ± 0.032	0.695
1-18	0.524 ± 0.061	0.747 ± 0.031	0.701
1-35	0.573 ± 0.059	0.724 ± 0.013	0.791
1-6	0.304 ± 0.061	0.767 ± 0.011	0.396
2-49	0.625 ± 0.039	0.742 ± 0.021	0.842
2-7	0.649 ± 0.034	0.708 ± 0.027	0.917
3-1	0.478 ± 0.046	0.763 ± 0.012	0.626
3-10	0.535 ± 0.044	0.747 ± 0.026	0.716
4-14	0.447 ± 0.093	0.758 ± 0.010	0.590
4-152	0.651 ± 0.034	0.754 ± 0.015	0.863
4-154	0.624 ± 0.053	0.778 ± 0.013	0.802
4-17	0.627 ± 0.053	0.746 ± 0.033	0.840
4-38	0.542 ± 0.059	0.724 ± 0.038	0.749
4-44-4	0.390 ± 0.101	0.750 ± 0.022	0.520
4-45	0.647 ± 0.039	0.781 ± 0.031	0.828
4-52	0.700 ± 0.046	0.731 ± 0.036	0.958
4-52-2	0.701 ± 0.017	0.753 ± 0.023	0.931
4-56	0.329 ± 0.109	0.769 ± 0.037	0.428
4-57	0.519 ± 0.057	0.794 ± 0.022	0.654
4-6	0.520 ± 0.042	0.705 ± 0.074	0.738
4-63	0.285 ± 0.075	0.744 ± 0.022	0.383
4-76	0.310 ± 0.102	0.768 ± 0.016	0.404
4-77	0.576 ± 0.130	0.766 ± 0.030	0.752
5-28	0.685 ± 0.044	0.766 ± 0.020	0.894
5-47	0.570 ± 0.068	0.748 ± 0.021	0.762
5-62	0.246 ± 0.042	0.731 ± 0.008	0.337
5-72	0.544 ± 0.057	0.729 ± 0.040	0.746
5-81	0.479 ± 0.044	0.774 ± 0.025	0.619
FYWM 3	0.694 ± 0.032	0.739 ± 0.021	0.939
FYWM 7	0.690 ± 0.026	0.746 ± 0.031	0.925
FZ-0	0.340 ± 0.107	0.690 ± 0.054	0.493
FZ-1	0.455 ± 0.068	0.745 ± 0.043	0.611
FZ-2	0.604 ± 0.012	0.731 ± 0.029	0.826
JK 2	0.409 ± 0.066	0.739 ± 0.031	0.553
PBZY	0.540 ± 0.087	0.733 ± 0.034	0.737
Z-7	0.642 ± 0.098	0.764 ± 0.027	0.840
ZNB	0.579 ± 0.069	0.773 ± 0.021	0.749
Fuding	0.550 ± 0.084	0.761 ± 0.029	0.723
Fuwuming	0.633 ± 0.056	0.759 ± 0.026	0.834
HJY	0.596 ± 0.051	0.730 ± 0.021	0.816
Jinxuan	0.467 ± 0.098	0.720 ± 0.012	0.649
Xiangshan 3	0.597 ± 0.047	0.760 ± 0.013	0.786
Xiangshan 5	0.475 ± 0.022	0.733 ± 0.013	0.648
Zhenghe	0.501 ± 0.076	0.732 ± 0.015	0.684
Zijuan	0.331 ± 0.138	0.725 ± 0.044	0.457
Mean	0.526 *	0.745	0.706

^1^ R_Fv/Fm_: The Fv/Fm ratio of the frozen group to the control group, * Being significantly different from that in control leaves at *p* < 0.001.

**Table 3:** Effects of freezing on indicators of Hunter color scales.

Color Indicator	*L*		*a*		*b*	
Treatment	Control	Frozen	Control	Frozen	Control	Frozen
1-10	20.69 ± 2.20	21.83 ± 3.33	−11.09 ± 0.63	−8.09 ± 0.11	32.33 ± 1.69	30.18 ± 1.77
1-16	21.27 ± 1.17	22.08 ± 1.79	−1.1 ± 0.40	−5.13 ± 1.00	33.44 ± 0.83	34.75 ± 3.14
1-17	24.23 ± 2.09	24.76 ± 2.66	−12.25 ± 0.54	−7.25 ± 1.66	40.74 ± 3.47	38.12 ± 4.29
1-18	21.41 ± 1.46	23.55 ± 3.07	−10.98 ± 0.72	−5.40 ± 1.00	36.27 ± 2.40	36.68 ± 2.89
1-35	24.05 ± 2.01	21.23 ± 3.46	−11.78 ± 0.28	−8.39 ± 1.27	38.53 ± 4.38	34.22 ± 4.28
1-6	25.96 ± 3.67	22.94 ± 2.10	−11.78 ± 0.96	−4.86 ± 1.48	43.07 ± 5.43	36.28 ± 2.82
2-49	19.14 ± 1.14	21.41 ± 5.00	−12.24 ± 0.77	−6.59 ± 1.64	31.28 ± 1.59	30.83 ± 4.72
2-7	22.37 ± 2.47	23.53 ± 2.63	−11.31 ± 0.75	−7.38 ± 0.57	37.20 ± 3.66	36.60 ± 2.70
3-1	21.52 ± 2.14	23.11 ± 0.81	−12.88 ± 0.27	−5.66 ± 0.92	35.93 ± 3.15	36.06 ± 0.63
3-10	21.27 ± 1.64	18.42 ± 1.02	−11.46 ± 0.34	−7.35 ± 0.35	35.23 ± 2.52	29.88 ± 1.47
4-14	17.40 ± 1.59	22.44 ± 2.36	−11.14 ± 0.35	−4.97 ± 0.40	29.58 ± 2.63	35.12 ± 2.54
4-152	20.45 ± 2.43	23.12 ± 1.60	−12.50 ± 0.53	−7.09 ± 1.24	34.01 ± 3.82	35.32 ± 6.64
4-154	25.39 ± 2.55	27.62 ± 2.98	−11.41 ± 0.62	−6.04 ± 1.02	41.37 ± 3.86	39.51 ± 3.48
4-17	21.55 ± 0.93	22.38 ± 0.68	−11.09 ± 0.95	−7.70 ± 1.34	34.32 ± 2.18	33.69 ± 2.40
4-38	23.09 ± 1.54	23.29 ± 1.12	−11.42 ± 0.32	−6.73 ± 0.23	38.62 ± 2.55	36.08 ± 2.94
4-44-4	22.69 ± 3.32	23.43 ± 1.20	−12.43 ± 0.51	−5.82 ± 1.22	35.79 ± 5.91	35.11 ± 2.46
4-45	19.75 ± 1.68	23.18 ± 1.94	−11.93 ± 0.67	−5.69 ± 1.08	31.05 ± 3.35	34.17 ± 3.66
4-52	21.56 ± 3.14	24.77 ± 2.97	−12.96 ± 0.40	−10.72 ± 0.39	32.45 ± 3.53	29.27 ± 1.45
4-52-2	23.21 ± 2.19	23.93 ± 3.42	−12.71 ± 0.45	−8.43 ± 1.07	32.20 ± 1.98	34.44 ± 5.17
4-56	19.91 ± 1.90	23.59 ± 3.53	−12.02 ± 0.61	−4.38 ± 1.23	33.49 ± 2.94	34.69 ± 4.89
4-57	26.29 ± 0.90	25.41 ± 3.21	−12.25 ± 1.47	−6.33 ± 1.07	43.11 ± 0.86	37.86 ± 4.93
4-6	21.42 ± 1.06	20.76 ± 1.71	−12.08 ± 0.50	−4.87 ± 1.17	32.20 ± 3.44	30.72 ± 3.68
4-63	22.50 ± 2.38	21.74 ± 2.11	−12.60 ± 0.49	−3.85 ± 0.28	37.52 ± 3.55	33.06 ± 5.56
4-76	19.71 ± 2.91	24.55 ± 2.60	−12.30 ± 0.89	−4.72 ± 0.65	33.04 ± 4.50	35.75 ± 4.41
4-77	21.90 ± 1.37	24.22 ± 2.84	−13.01 ± 1.08	−12.16 ± 1.25	34.39 ± 2.17	37.74 ± 4.89
5-28	22.95 ± 2.02	22.00 ± 1.22	−11.90 ± 0.34	−7.71 ± 1.19	35.77 ± 2.64	33.96 ± 3.19
5-47	22.49 ± 1.80	22.38 ± 2.42	−12.79 ± 0.38	−5.92 ± 0.99	37.98 ± 2.93	35.68 ± 3.23
5-62	22.20 ± 2.34	20.67 ± 3.29	−11.43 ± 0.79	2.48 ± 1.91	34.03 ± 1.94	31.05 ± 1.94
5-72	21.52 ± 1.98	23.15 ± 1.26	−11.23 ± 0.45	−6.18 ± 0.96	34.13 ± 3.02	36.89 ± 2.02
5-81	21.47 ± 2.38	20.20 ± 2.21	−12.84 ± 0.75	−9.89 ± 1.01	33.32 ± 1.89	30.58 ± 2.42
FYWM3	20.29 ± 2.06	22.94 ± 2.73	-11.65 ± 0.87	−9.31 ± 0.44	34.38 ± 3.37	36.93 ± 2.65
FYWM7	24.44 ± 2.34	20.89 ± 1.34	−10.56 ± 0.85	−8.88 ± 0.94	32.54 ± 3.88	32.75 ± 0.72
FZ-0	26.53 ± 0.97	27.34 ± 2.91	−10.84 ± 0.29	−3.87 ± 1.97	43.73 ± 1.80	41.35 ± 1.53
FZ-1	18.08 ± 0.51	20.76 ± 1.57	−11.15 ± 0.48	−4.8 ± 0.81	30.64 ± 0.72	33.29 ± 2.72
FZ-2	22.19 ± 1.09	23.61 ± 1.14	−12.48 ± 0.62	−8.35 ± 0.87	37.27 ± 1.72	37.60 ± 1.70
JK-2	24.97 ± 2.29	28.29 ± 3.87	−12.81 ± 0.61	−4.79 ± 0.88	39.31 ± 4.67	35.04 ± 2.83
PBZY	19.29 ± 2.05	23.30 ± 2.33	−11.48 ± 0.34	−6.21 ± 0.63	31.48 ± 2.42	28.66 ± 8.03
Z-7	21.95 ± 2.22	23.13 ± 0.56	−11.49 ± 1.01	−9.86 ± 2.12	36.18 ± 4.06	35.65 ± 2.07
ZNB	20.56 ± 0.80	22.17 ± 1.38	−12.46 ± 0.37	−7.84 ± 1.17	33.49 ± 1.89	31.98 ± 4.45
Fuding	20.77 ± 1.92	24.26 ± 2.43	−11.94 ± 0.53	−6.82 ± 1.18	34.95 ± 2.76	30.20 ± 6.12
Fuwuming	26.96 ± 1.96	30.77 ± 3.99	−13.24 ± 1.68	−7.14 ± 1.66	40.41 ± 3.33	41.88 ± 5.85
HJY	54.26 ± 11.34	43.60 ± 5.68	−1.79 ± 5.67	5.98 ± 2.95	59.5 ± 11.79	50.25 ± 2.68
Jinxuan	21.22 ± 3.27	20.28 ± 1.82	−10.12 ± 0.70	−7.06 ± 0.48	35.82 ± 5.58	33.69 ± 3.14
Xiangshan 3	18.85 ± 1.58	24.94 ± 4.64	−11.56 ± 0.66	−7.42 ± 0.55	30.70 ± 2.24	34.99 ± 5.59
Xiangshan 5	18.55 ± 1.01	19.87 ± 1.35	−12.21 ± 0.55	−5.98 ± 1.4	31.52 ± 1.69	32.38 ± 2.05
Zhenghe	22.83 ± 2.11	22.48 ± 1.63	−10.9 ± 0.85	−7.25 ± 0.66	29.40 ± 2.60	31.51 ± 3.42
Zijuan	23.19 ± 1.76	25.74 ± 1.59	−12.21 ± 0.42	−2.44 ± 0.81	37.45 ± 2.26	35.41 ± 2.64
Mean	22.64	23.62 *	−11.66	−6.27 **	35.77	34.85 *

* Being significantly different from that of the control group at *p* < 0.05, ** Being significantly different from that of the control group at *p* < 0.01.

## Supplementary Materials

The following are available online at https://www.mdpi.com/2223-7747/8/10/434/s1, Figure S1: Monthly changes in maximum (upper) and minimum (lower) temperature during 2011–2018 in Hangzhou; Figure S2: Daily changes in maximum (blue line) and minimum (yellow line) temperature during February 2016 in Hangzhou; Figure S3: Effect of freezing on leaf color of various tea cultivars, Table S1: The linear correlation and regressive relationship between various tested indicators; Table S2: Freezing-sensitive score based on freezing-induced injured leaves on a plant; Table S3: Freezing tolerance classification of tea cultivars based on H index; Table S4: The rt-qPCR primers used in the test.
