# Supplementary material for: Effect of Freezing on Photosystem II and Assessment of Freezing Tolerance of Tea Cultivar
Source: Plants (Basel). 2019 Oct 22;8(10):434. doi: 10.3390/plants8100434 (PMC6843692; doi:10.3390/plants8100434)
Supplement: Supplementary file 1 [file plants-08-00434-s001.zip › sulpplemetary for conversion/Figure S1 (R2).docx]

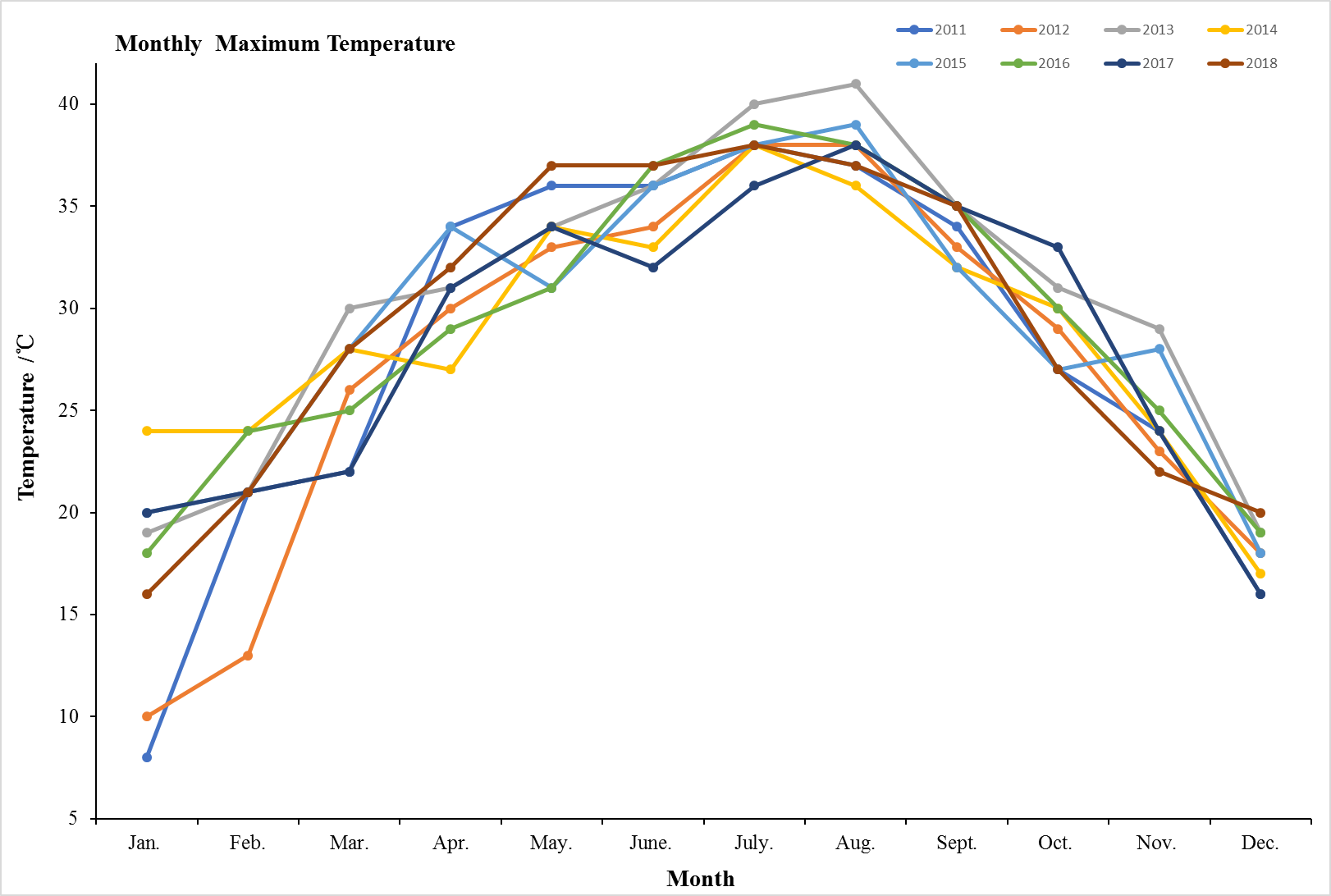


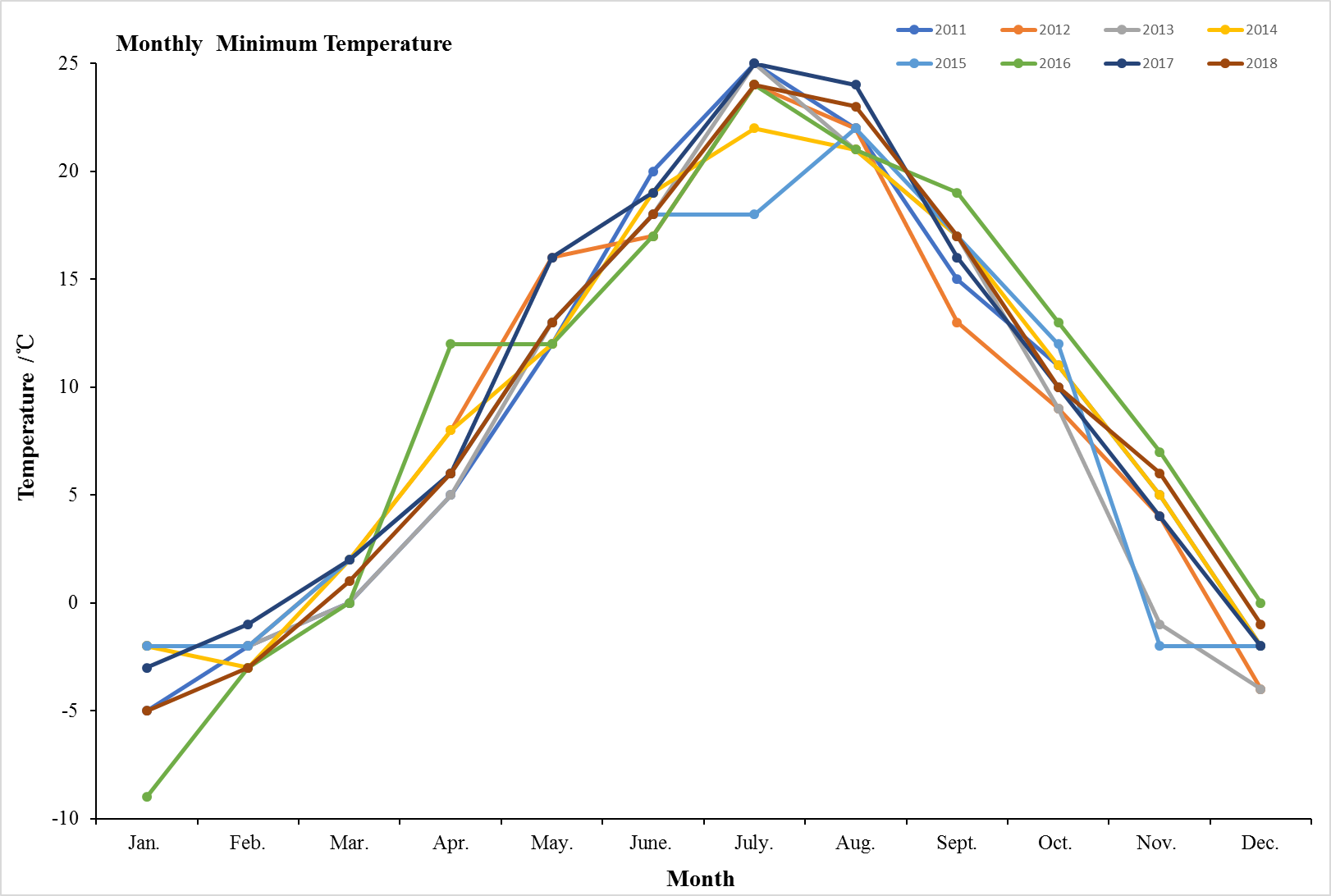


Figure S1. Monthly changes in maximum (upper) and minimum (lower) temperature during 2011-2018 in Hangzhou. The data was collected from “Tianqihoubao” database (http://www.tianqihoubao.com/lishi/hangzhou.html).
