# Supplementary material for: Effect of Freezing on Photosystem II and Assessment of Freezing Tolerance of Tea Cultivar
Source: Plants (Basel). 2019 Oct 22;8(10):434. doi: 10.3390/plants8100434 (PMC6843692; doi:10.3390/plants8100434)
Supplement: Supplementary file 1 [file plants-08-00434-s001.zip › sulpplemetary for conversion/Figure S3 (R2).docx]

| 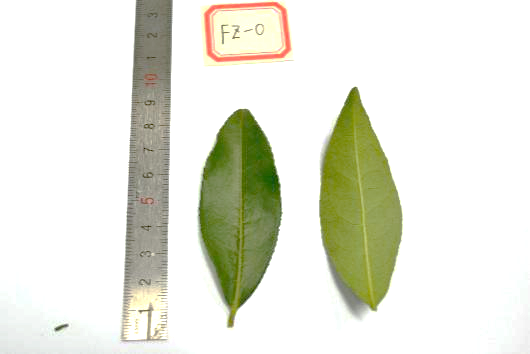 | 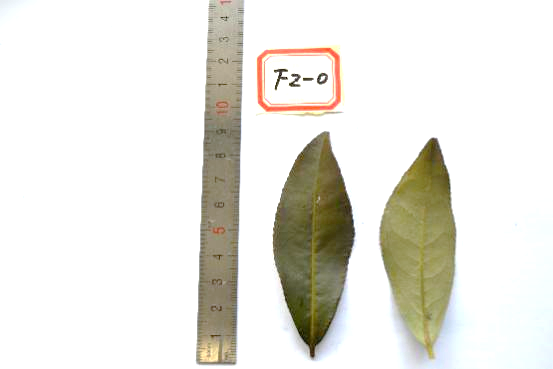 |
| --- | --- |
| Cultivar ‘FZ-0’ control | Cultivar ‘FZ-0’ chilled |
| 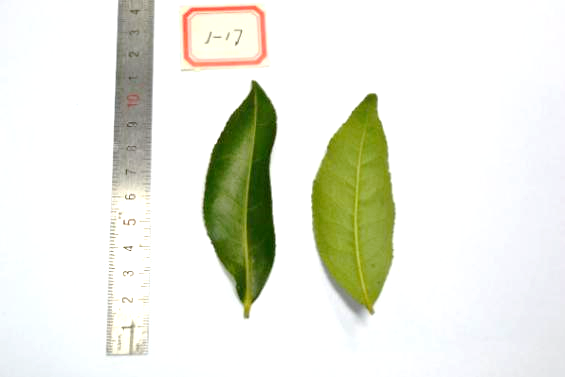 | 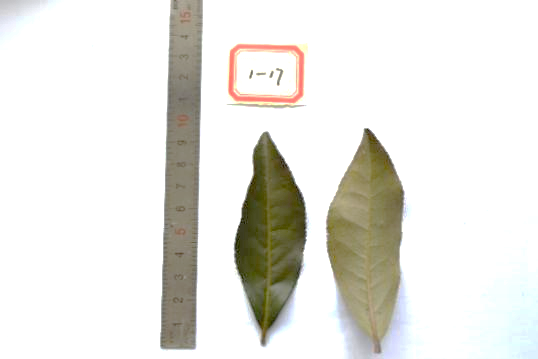 |
| Cultivar ‘1-17’ control | Cultivar ‘1-17’ chilled |
| 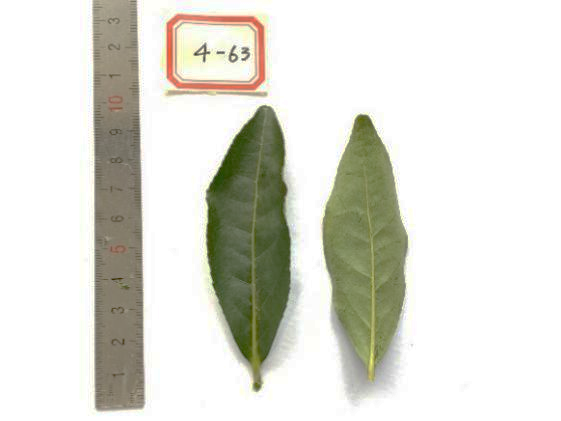 | 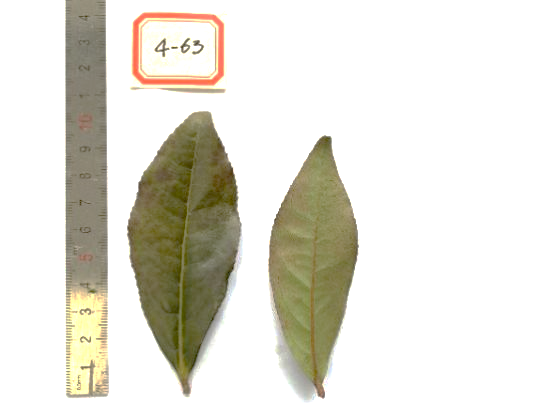 |
| Cultivar ‘4-63’ control | Cultivar ‘4-63’ chilled |
| 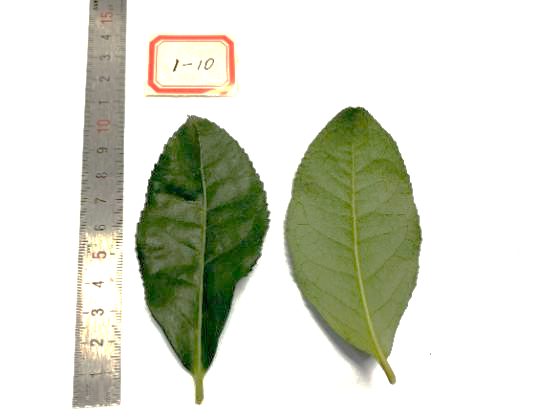 | 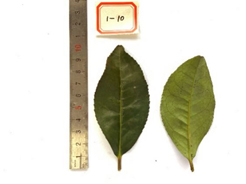 |
| Cultivar ‘1-10’ control | Cultivar ‘1-10’ chilled |

Figure S3 Effect of freezing on leaf color of various tea cultivars

Tea shoots with an apex bud and six leaves were frozen at -15°C for 2 hours, and then placed in a 500-mL beaker containing 200 mL purified water at 20°C for 5 hours. The showed leaf was fourth leaf beneath apex bud.
