# Supplementary material for: Effect of Freezing on Photosystem II and Assessment of Freezing Tolerance of Tea Cultivar
Source: Plants (Basel). 2019 Oct 22;8(10):434. doi: 10.3390/plants8100434 (PMC6843692; doi:10.3390/plants8100434)
Supplement: Supplementary file 1 [file plants-08-00434-s001.zip › sulpplemetary for conversion/Table S 1ú¿R2).docx]

Table S1. The linear correlation and regressive relationship between various tested indicators ^a^

| Cultivars | H | R_Fv/Fm_ | R*_L_* | R*_a_* | R*_b_* |
| --- | --- | --- | --- | --- | --- |
| 1-10 | 0.00 | 0.877 | 1.055 | 0.729 | 0.933 |
| 1-16 | 28.33 | 0.534 | 1.038 | 0.462 | 1.039 |
| 1-17 | 48.33 | 0.695 | 1.022 | 0.592 | 0.936 |
| 1-18 | 23.33 | 0.701 | 1.100 | 0.492 | 1.011 |
| 1-35 | 1.67 | 0.791 | 0.883 | 0.712 | 0.888 |
| 1-6 | 28.33 | 0.396 | 0.884 | 0.413 | 0.842 |
| 2-49 | 25.00 | 0.842 | 1.119 | 0.538 | 0.986 |
| 2-7 | 25.00 | 0.917 | 1.052 | 0.653 | 0.984 |
| 3-1 | 25.00 | 0.626 | 1.074 | 0.439 | 1.004 |
| 3-10 | 25.00 | 0.716 | 0.866 | 0.641 | 0.848 |
| 4-14 | 26.67 | 0.59 | 1.290 | 0.446 | 1.187 |
| 4-152 | 25.00 | 0.863 | 1.131 | 0.567 | 1.039 |
| 4-154 | 25.00 | 0.802 | 1.088 | 0.529 | 0.955 |
| 4-17 | 25.00 | 0.84 | 1.039 | 0.694 | 0.982 |
| 4-38 | 25.00 | 0.749 | 1.009 | 0.589 | 0.934 |
| 4-44-4 | 25.00 | 0.52 | 1.033 | 0.468 | 0.981 |
| 4-45 | 25.00 | 0.828 | 1.174 | 0.477 | 1.100 |
| 4-52 | 0.00 | 0.958 | 1.149 | 0.827 | 0.902 |
| 4-52-2 | 0.00 | 0.931 | 1.031 | 0.663 | 1.070 |
| 4-56 | 50.00 | 0.428 | 1.185 | 0.364 | 1.036 |
| 4-57 | 48.33 | 0.654 | 0.967 | 0.517 | 0.878 |
| 4-6 | 25.00 | 0.738 | 0.969 | 0.403 | 0.954 |
| 4-63 | 33.33 | 0.383 | 0.966 | 0.306 | 0.881 |
| 4-76 | 25.00 | 0.404 | 1.246 | 0.384 | 1.082 |
| 4-77 | 0.00 | 0.752 | 1.106 | 0.935 | 1.097 |
| 5-28 | 18.33 | 0.894 | 0.959 | 0.648 | 0.949 |
| 5-47 | 25.00 | 0.762 | 0.995 | 0.463 | 0.939 |
| 5-62 | 50.00 | 0.337 | 0.931 | -0.217 | 0.912 |
| 5-72 | 25.00 | 0.746 | 1.076 | 0.550 | 1.081 |
| 5-81 | 25.00 | 0.619 | 0.941 | 0.770 | 0.918 |
| FYWM 3 | 0.00 | 0.939 | 1.131 | 0.799 | 1.074 |
| FYWM 7 | 0.00 | 0.925 | 0.855 | 0.841 | 1.006 |
| FZ-0 | 50.00 | 0.493 | 1.031 | 0.357 | 0.946 |
| FZ-1 | 25.00 | 0.611 | 1.148 | 0.430 | 1.086 |
| FZ-2 | 25.00 | 0.826 | 1.064 | 0.669 | 1.009 |
| JK 2 | 28.33 | 0.553 | 1.133 | 0.374 | 0.891 |
| PBZY | 25.00 | 0.737 | 1.208 | 0.541 | 0.910 |
| Z-7 | 20.00 | 0.84 | 1.054 | 0.858 | 0.985 |
| ZNB | 25.00 | 0.749 | 1.078 | 0.629 | 0.955 |
| Fuding | 25.00 | 0.723 | 1.168 | 0.571 | 0.864 |
| Fuwuming | 25.00 | 0.834 | 1.141 | 0.539 | 1.036 |
| HJY | 50.00 | 0.816 | 0.804 | -3.341 | 0.845 |
| Jinxuan | 25.00 | 0.649 | 0.956 | 0.698 | 0.941 |
| Xiangshan 3 | 25.00 | 0.786 | 1.323 | 0.642 | 1.140 |
| Xiangshan 5 | 25.00 | 0.648 | 1.071 | 0.490 | 1.027 |
| Zhenghe | 25.00 | 0.684 | 0.985 | 0.665 | 1.072 |
| Zijuan | 43.33 | 0.457 | 1.110 | 0.200 | 0.946 |
| r ^b^ |  | -0.610  (p<0.01) | -0.066 (p>0.05) | -0.480  (p<0.01) | -0.242 (p>0.05) |
| Regressive relationship |  | H=60.305-50.086R_Fv/Fm_ |  | H=30.033-10.821R*a* |  |

^a^: H: Freezing tolerance index; R_Fv/Fm_: The ratio of the frozen group Fv/Fm to the control group Fv/Fm; R_L_: The ratio of the frozen group Hunter color scale *L* to the control group Hunter color scale *L*; R_a_: The ratio of the frozen group Hunter color scale *a* to the control group Hunter color scale *a*; R_b_: The ratio of the frozen group Hunter color scale *b* to the control group Hunter color scale *b.*

^b^: r: Pearson’s linear correlation coefficient.
