# Supplementary material for: Effect of Freezing on Photosystem II and Assessment of Freezing Tolerance of Tea Cultivar
Source: Plants (Basel). 2019 Oct 22;8(10):434. doi: 10.3390/plants8100434 (PMC6843692; doi:10.3390/plants8100434)
Supplement: Supplementary file 1 [file plants-08-00434-s001.zip › sulpplemetary for conversion/Table S 2 (R2).docx]

Table S2. Freezing-sensitive score based on freezing-induced injured leaves on a plant ^a^

| Grading score (i) | 0 | 1 | 2 | 3 | 4 |
| --- | --- | --- | --- | --- | --- |
| Injured leaves (%) | ≦5.0 | 5.0 ~≦15.0 | 15.0 ~≦25.0 | 25.0 ~≦50.0 | >50.0 |

^a.^Field experiment was random block design, with 3 blocks for each cultivar and 15 plants each block; injured leaves and total leaves on 5 plants each block were investigated.
