# Supplementary material for: Effect of Freezing on Photosystem II and Assessment of Freezing Tolerance of Tea Cultivar
Source: Plants (Basel). 2019 Oct 22;8(10):434. doi: 10.3390/plants8100434 (PMC6843692; doi:10.3390/plants8100434)
Supplement: Supplementary file 1 [file plants-08-00434-s001.zip › sulpplemetary for conversion/Table S 3 (R2).docx]

Table S3. Freezing tolerance classification of tea cultivars based on H index ^a^

| Freezing tolerance | Highly freezing-tolerant | Freezing-tolerant | Moderate sensitive | Freezing sensitive |
| --- | --- | --- | --- | --- |
| H | < 10.0 | 10.0≦H<20.0 | 20.0≦H<50.0 | ≧50.0 |

^a.^H index was calculated as described in Section 4.2.
