# Supplementary material for: Effect of Freezing on Photosystem II and Assessment of Freezing Tolerance of Tea Cultivar
Source: Plants (Basel). 2019 Oct 22;8(10):434. doi: 10.3390/plants8100434 (PMC6843692; doi:10.3390/plants8100434)
Supplement: Supplementary file 1 [file plants-08-00434-s001.zip › sulpplemetary for conversion/Table S 4 (R2).docx]

Table S2. The rt-qPCR primers used in the test ^a^

| Gene ID | Primer Sequence | |
| --- | --- | --- |
| *β-actin* | Forward Primer | CTTCCTCATGCTATCCTCCGTCTT |
|  | Reverse Primer | ATTTCCCGTTCAGCAGTGGTG |
| *psbA* | Forward Primer | CAGATTCGGTCAAGAGGAAGAA |
|  | Reverse Primer | CCAAGCAGCCAAGAAGAAATG |
| *psbD* | Forward Primer | CGCAGTTTCTACTCCTGCTAAT |
|  | Reverse Primer | AAGTCCACAGACCACCTAATTG |

^a.^The rt-PCR primers for *psbA* (genome ID: TEA001596.1) and *psbD* (genome ID: TEA011225.1) were designed according to published reference genome [49]. The primer for internal control β-actin gene was designed according to published sequence [50].
